# Cyclooxygenase-2 Selectively Controls Renal Blood Flow Through a Novel PPARβ/δ-Dependent Vasodilator Pathway

**DOI:** 10.1161/HYPERTENSIONAHA.117.09906

**Published:** 2018-02-07

**Authors:** Nicholas S. Kirkby, Walkyria Sampaio, Gisele Etelvino, Daniele T. Alves, Katie L. Anders, Rafael Temponi, Fisnik Shala, Anitha S. Nair, Blerina Ahmetaj-Shala, Jing Jiao, Harvey R. Herschman, Xiaomeng Wang, Walter Wahli, Robson A. Santos, Jane A. Mitchell

**Affiliations:** From the Vascular Biology, National Heart and Lung Institute, Imperial College London, United Kingdom (N.S.K., K.L.A., F.S., A.S.N., B.A.-S., J.A.M.); Department of Physiology and Biophysics, Federal University of Minas Gerais, Belo Horizonte, Brazil (W.S., G.E., D.T.A., R.T., R.A.S.); Department of Medical and Molecular Pharmacology, David Geffen School of Medicine, University of California, Los Angeles (J.J., H.R.H.); Vascular Biology Laboratory, Lee Kong Chian School of Medicine (X.W.) and Lee Kong Chian School of Medicine (W.W), Nanyang Technological University, Singapore, Singapore; Institute of Molecular and Cell Biology, Proteos, Agency for Science Technology and Research, Singapore, Singapore (X.W.); Department of Cell Biology, Institute of Ophthalmology, University College London, United Kingdom (X.W.); Singapore Eye Research Institute (X.W.); and Center for Integrative Genomics, University of Lausanne, Switzerland (W.W.).

**Keywords:** cyclooxygenase 2, endothelium, inflammation, regional blood flow, spleen

## Abstract

Cyclooxygenase-2 (COX-2) is an inducible enzyme expressed in inflammation and cancer targeted by nonsteroidal anti-inflammatory drugs. COX-2 is also expressed constitutively in discreet locations where its inhibition drives gastrointestinal and cardiovascular/renal side effects. Constitutive COX-2 expression in the kidney regulates renal function and blood flow; however, the global relevance of the kidney versus other tissues to COX-2–dependent blood flow regulation is not known. Here, we used a microsphere deposition technique and pharmacological COX-2 inhibition to map the contribution of COX-2 to regional blood flow in mice and compared this to COX-2 expression patterns using luciferase reporter mice. Across all tissues studied, COX-2 inhibition altered blood flow predominantly in the kidney, with some effects also seen in the spleen, adipose, and testes. Of these sites, only the kidney displayed appreciable local COX-2 expression. As the main site where COX-2 regulates blood flow, we next analyzed the pathways involved in kidney vascular responses using a novel technique of video imaging small arteries in living tissue slices. We found that the protective effect of COX-2 on renal vascular function was associated with prostacyclin signaling through PPARβ/δ (peroxisome proliferator-activated receptor-β/δ). These data demonstrate the kidney as the principle site in the body where local COX-2 controls blood flow and identifies a previously unreported PPARβ/δ-mediated renal vasodilator pathway as the mechanism. These findings have direct relevance to the renal and cardiovascular side effects of drugs that inhibit COX-2, as well as the potential of the COX-2/prostacyclin/PPARβ/δ axis as a therapeutic target in renal disease.

## Abstract 2

Supplemental Digital Content is available in the text.

Cyclooxygenase-2 (COX-2) is an inducible enzyme expressed at sites of inflammation and in cancer. As such, COX-2 is the therapeutic target for nonsteroidal anti-inflammatory drugs (NSAID) that include ibuprofen, diclofenac, and celecoxib. NSAIDs are among the world’s most consumed medications to treat pain and inflammation, with an estimated 30 billion doses consumed annually in the United States.^1^ NSAIDs can also prevent cancer with prospective clinical trials reporting that celecoxib prevents ≈50% of colon cancers in susceptible individuals.^2^ The greatest limitation in the therapeutic use of NSAIDs is their cardiovascular and renal safety profile. Indeed, NSAIDs are an independent risk factor for cardiovascular events, and concern surrounding their cardiovascular side effects has virtually arrested new drug development and led to a failure to realize the full potential of blocking COX-2 for cancer prevention. Thus, there is an urgent need to understand how COX-2 protects the cardiovascular system. It was initially thought that cardiovascular side effects were limited to drugs that selectively target COX-2,^3,4^ such as Vioxx (rofecoxib) and Celebrex (celecoxib), which were introduced in the early 2000s.^5^ However, as a result of subsequent epidemiology analyses^6^ and 2 large recent clinical cardiovascular outcome studies, SCOT (Standard Care Versus Celecoxib Outcome Trial)^7^ and PRECISION (Prospective Randomized Evaluation of Celecoxib Integrated Safety vs Ibuprofen or Naproxen),^8^ it is clear that traditional NSAIDs, including ibuprofen, carry at least as great a cardiovascular and renal risk as the COX-2 selective drug celecoxib.

The mechanisms by which NSAIDs produce their cardiovascular side effects remain hotly debated, but underlying causes include increases in thrombosis, atherosclerosis, and blood pressure. There is agreement in the field that cardiovascular side effects of NSAIDs involve loss of key protective downstream prostaglandins.^9,10^ These include (1) prostacyclin, a vasodilator and antiplatelet hormone that acts via classical cell surface I-prostanoid (IP) receptors to increase cAMP and the nuclear receptor PPARβ/δ (peroxisome proliferator-activated receptor-β/δ) to signal with both genomic and nongenomic pathways and (2) prostaglandin E_2_ (PGE_2_) that can also cause vasodilation through cAMP via EP2 and EP4 receptors.^11^ There is also agreement in the field that cardiovascular side effects of NSAIDs occur via inhibition of constitutively expressed COX-2 because the relative risk is the same (1.3) in those with cardiovascular/inflammatory disease as in people without systemic inflammatory disease (eg, healthy individuals or patients with osteoarthritis or at risk of colon cancer) and can be modeled in healthy mice^9,10^ in the absence of local or systemic inflammation. What remains contentious is the precise site of cardioprotective constitutive COX-2. Is it throughout the systemic endothelium as some suggest^10^ or is it in the kidney?^12^ The case for pan-endothelial COX-2 is supported by studies measuring urinary levels of the prostacyclin metabolite, PGIM (prostaglandin I-metabolite [2,3-dinor-6-keto-prostaglandin F1α]) , and tissue-specific COX-2 knockout mice. However, generalized endothelial COX-2 expression is not present in healthy blood vessels from humans^13^ or mice,^14–16^ and PGIM can originate from the kidney.^17^ The case for a renal COX-2 is supported by a clear body of evidence showing COX-2 is constitutively expressed in the kidney^12^ via NFAT (nuclear factor of activated T-cells)-mediated transcription^18^ and the contribution of renal COX-2 to regulation of (1) blood pressure,^12^ (2) the renin–angiotensin system, and (3) specific vasomotor gene pathways, including the endogenous NO synthase inhibitor ADMA (asymmetric dimethylarginine).^9^ In line with this, NSAIDs also reduce renal blood flow as a direct result of blocking COX-2.^12,19,20^ Indeed, it has been suggested that, on a population basis, the magnitude of blood pressure^21^ or circulating ADMA elevation^9^ produced by COX-2 inhibitors is entirely sufficient to explain the cardiovascular side effects produced by NSAIDs.

Given the importance of constitutive COX-2 to our understanding of the cardiovascular and renal side effects of NSAIDs, in the present study, we have addressed its role in the control of regional blood flow for a wide range of tissue sites, including the kidney. We have done this by determining the effect of acute COX-2 inhibition on local regional blood flow throughout the body and mapped this to the distribution of COX-2 across the same tissues.

## Methods

The authors declare that all supporting data are available within the article and its online-only Data Supplement. A full methodology is provided in the online-only Data Supplement. Briefly, regional blood flow was measured in wild-type mice using a microsphere deposition method,^22^ before and 20 minutes after administration of the selective COX-2 inhibitor, parecoxib (5 mg/kg; IV; Pfizer). In parallel, COX-2 expression was mapped using bioluminescent imaging of tissue from COX-2 luciferase reporter mouse (*Cox2*^fLuc/+^).^23^ Kidney and spleen prostaglandin, cAMP, and cGMP content were measured in homogenates by immunoassay and gene expression by quantitative polymerase chain reaction. Vascular responses were measured by wire myography, and in small intrarenal arcuate arteries, using a novel technique of video imaging in live precision-cut kidney slices based on early studies using lung.^24,25^ Experiments were performed on male, 8-week-old mice after ethical review and in accordance with local/national guidelines.

### Statistics and Data Analysis

Data are presented as means±SE for n experiments. Data were statistically analyzed using Prism 7 software (Graphpad Software) as defined in figure legends—typically either Student unpaired *t* test or 2-way ANOVA—and considered significant where *P*<0.05.

## Results and Discussion

### Regulation of Regional Blood Flow In Vivo by Constitutive COX-2

**Figure 1.**
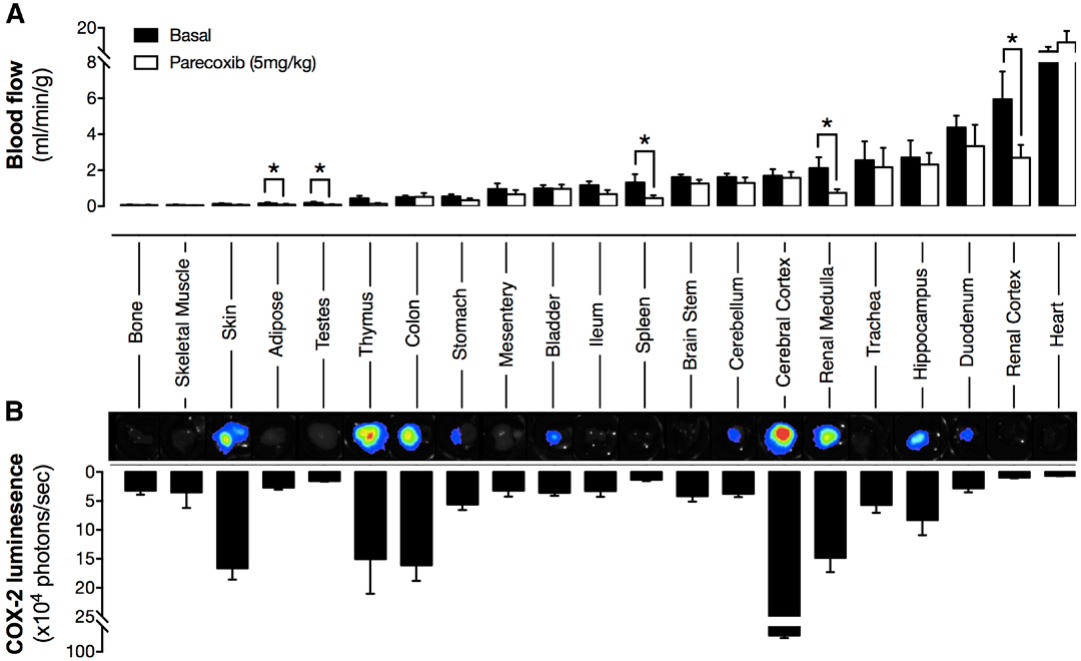
Effect of cyclooxygenase-2 (COX-2) inhibition on regional blood flow (**A**) and level of constitutive enzyme expression (**B**). Blood flow was measured using a microsphere deposition technique basally and after COX-2 inhibition by parecoxib (5 mg/kg IV). COX-2 expression was measured by bioluminescent imaging of tissue from *Cox2*^fLuc/+^ mice. Inset panels show representative images with luminescent signal with the scale red (highest) > black (lowest). Data are presented as mean±SE. *P<0.05 by Student unpaired *t* test. n=5 to 6.

In this study, we applied the microsphere deposition technique of regional blood flow measurement^22,26,27^ to determine in an unbiased way the relative contribution of constitutive COX-2 to vascular control in the kidney compared with the rest of the body (Figure 1A). To do this, we chose a model of acute pharmacological COX-2 inhibition which allows the role of prostaglandin generation on vascular function to be evaluated without the confounding effects on renal function that long-term COX-2 inhibition causes. Although the role of COX-2 may vary in disease, we specifically chose to perform our experiments in healthy adult mice because this model has been used to mechanistically define the key cardioprotective functions of COX-2 in the absence of inflammation, including restraining thrombotic tone,^10^ and maintaining blood pressure and preserving endothelial and renal functions.^9^ Moreover, although COX-2 is readily induced in tissues by physiological stress^12^ or systemic inflammation^28^ (but only modestly in atherosclerosis^29^), cardiovascular side effects of NSAIDs occur in people with a range of varied disease pathogeneses, including colon cancer and osteoarthritis, that are not associated with systemic inflammation.^5,6^

**Figure 2.**
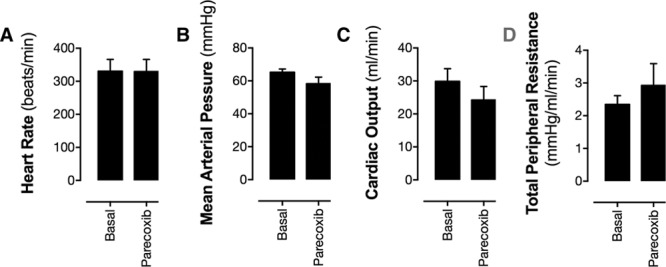
Effect of acute cyclooxygenase-2 inhibition on heart rate (**A**), mean arterial blood pressure (**B**), cardiac output (**C**), and total peripheral vascular resistance (**D**). Heart rate and blood pressure were measured by carotid artery cannulation, cardiac output from microsphere ejection rate, and total peripheral resistance calculated. Data are presented as mean±SE. *P<0.05 by Student unpaired *t* test. n=5 to 6.

At the dose used, parecoxib produced >90% COX-2 inhibition without detectable COX-1 inhibition when assessed by ex vivo bioassay (Figure S1 in the online-only Data Supplement). As has been shown before, blocking COX-2 activity with acute systemic parecoxib administration resulted in a clear and profound reduction in basal blood flow in both the renal medulla and cortex (Figure 1A). COX-2 inhibition also reduced blood flow in spleen, adipose tissue, and testes; however, in the majority of tissues, parecoxib had no effect on local blood flow (Figure 1A). In agreement with the localized nature of the changes in blood flow, parecoxib did not alter systemic blood pressure, heart rate, cardiac output, or total peripheral vascular resistance (Figure 2), which also supports the idea that blood flow reductions are because of loss of local COX-2–driven prostanoids and do not reflect changes in juxtaglomerular feedback-driven angiotensin II or other circulating pressors.

We next mapped regional COX-2–driven blood flow onto the pattern of constitutive COX-2 expression in tissues using *Cox2*^fLuc/+^ reporter mice. Although we have previously reported regional COX-2 expression in some tissues using this approach,^15,18^ here we have extended this array to match the full panel of structures where blood flow was studied (Figure 1B). As we have shown previously, constitutive COX-2 expression was observed in healthy tissue in the absence of inflammation in the renal medulla, skin, thymus, colon, and brain (Figure 1B). Notably, the strong expression in the renal medulla correlates closely with the powerful effect of COX-2 inhibition on blood flow in this region. However, in other tissues, no such overlap was seen. For example, although the brain and gut showed strong constitutive COX-2 expression, COX-2 inhibition produced no local change in blood flow at these sites indicating that the link between COX-2 expression and blood flow control is complex and perhaps reflects the degree of COX-2 activation, the cell types expressing the enzyme, and the prostanoids they produce. Conversely, and perhaps more surprisingly, in the renal cortex, spleen, adipose tissue, and testes, where blood flow was reduced by COX-2 inhibition, minimal local COX-2 expression was observed in luciferase reporter mice. In the case of the renal cortex, this may be explained by the vascular architecture of the kidney where blood enters the organ through the COX-2–rich medulla region, from where it may carry prostanoids into the cortex. In the spleen, adipose, and testes, however, the effect of parecoxib on blood flow is more difficult to explain. It is unlikely to reflect the sensitivity of the reporter mouse imaging technique used to detect COX-2 expression as we have previously demonstrated this approach has similar or greater sensitivity to antibody-based protein detection and activity measurement.^15,23^

### COX-2 Expression and Activity Within Tissues

**Figure 3.**
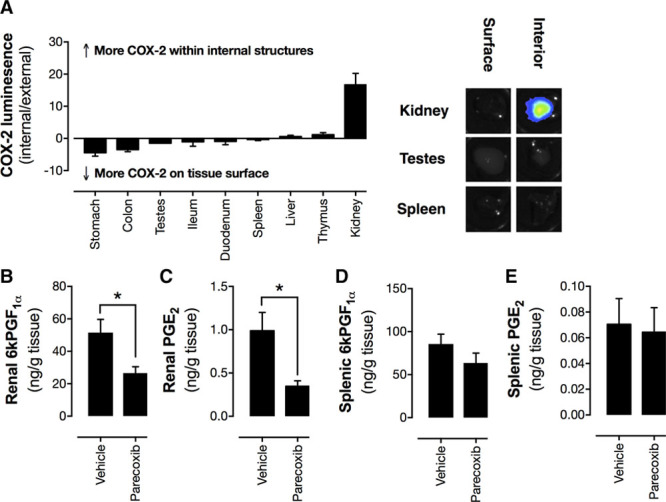
Level of cyclooxygenase-2 (COX-2) expression within tissues (**A**) and prostaglandin levels in homogenates of renal medulla (**B** and **C**) and spleen (**D** and **E**) from parecoxib-treated mice. COX-2 expression was measured by bioluminescent imaging of tissue from *Cox2*^fLuc/+^ mice. Inset panels show representative images with luminescent signal with the scale red (highest) > black (lowest). Prostacyclin (measured as 6 ketoPGF_1α_[6-keto prostaglandin F1α]) and prostaglandin E_2_ (PGE_2_) levels in homogenates. Data are presented as means±SE. *P<0.05 by Student unpaired *t* test. n=5 to 6.

In the case of the spleen and testes, solid tissues in which bioluminescence imaging may be limited by tissue penetrance, we reasoned that internal structures may express sufficient COX-2 to control local blood flow regulation. To explore this possibility, we performed a study using a range of solid tissues from *Cox2*^fLuc/+^ mice, in which luminescence was imaged from both the tissue surface (as in Figure 1) and in tissue bisected to reveal internal structures (Figure 3A); like we have done for the kidney and heart in our previous work. As we have shown previously,^15,18^ bisecting the kidney to reveal the medulla region produced ≈20-fold increase in detected *Cox2* promoter-driven bioluminescence. In spleen and testes, however, the effect of tissue division was negligible, indicating that in these tissues, COX-2 is essentially absent (Figure 3B).

To further exclude the possibility that these organs might contain local COX-2 activity undetectable by reporter mouse tissue imaging, we measured prostacyclin and PGE_2_, the best established vasodilator prostanoids, as functional readouts of COX-2 activity. We focused our analysis on the spleen and used the renal medulla as a positive control for a tissue with high constitutive COX-2 expression. Wild-type mice treated with parecoxib in vivo to block COX-2 activity had reduced prostacyclin and PGE_2_ in the renal medulla (Figure 3B and 3C), confirming the effectiveness of parecoxib and what we know of COX-2 expression in this tissue. By contrast, parecoxib had no effect on prostacyclin or PGE_2_ content in the spleen (Figure 3D and 3E). In this regard, the pathways regulating blood flow in the spleen, adipose, and testes remain unclear and beyond the scope of this investigation. One possibility is that regulation of blood flow in these regions is a function of COX-2 expressed in the brain. For example, in the spleen, it is known that blood flow is under tonic control by sympathetic activity and that conditions which increase central COX-2 expression increase splenic blood flow via neural activation.^30^

Taken together, these data demonstrate that the kidney is the only site in the body where local constitutive COX-2 expression impacts on vascular function. Our observation that COX-2 inhibition selectively effects the renal vasculature while having little to no effect on the majority of vascular beds adds to several existing lines of evidence to suggest the kidney rather than the systemic vasculature is the major site where constitutive COX-2 is locally active within the cardiovascular system.^14,15,18^ As such, it supports the idea that renal, rather than generalized endothelial/vascular, expression can for account the cardioprotective effects of constitutive COX-2. This, in turn, may help explain why COX-2 inhibition by NSAIDs is harmful to the cardiovascular system even in healthy mice and patients who do not have systemic inflammation. This applies not only to control of blood pressure and renal function but also in the absence of clear evidence that COX-2 is expressed and active in the systemic vasculature, inhibition of renal COX-2 may also provide an explanation for increase thrombotic events seen in people taking COX-2 inhibitors given the close links between blood pressure, circulating renal hormones, and systemic vascular disease as previously suggested.^9,21^

### Role of COX Isoforms and Prostanoids in Renal Arterioles In Situ

As the main site where COX-2 controls blood flow, we went on to explore the associated downstream mechanisms regulating renal vascular function. To do this, we devised a novel approach to measure responses of small arcuate arteries in situ, by video microscopy, in precision-cut organotypic kidney slices. The use of precision-cut organ slices, where cellular integrity and biological function are maintained, is routine in toxicological studies.^31^ Furthermore, our group and others have used video imaging to monitor real-time vascular responses in lung slices.^24,25^ However, to our knowledge, this is the first time this approach has been applied to renal vascular responses. Arcuate arteries are the smallest renal arterial vessels identifiable using this technique. Because of their position within the renal vascular tree, these vessels contribute both to renal blood flow regulation and glomerular filtration. We compared responses in these vessels to those in isolated segments of the main renal artery and aorta, for which we used traditional myography.

**Figure 4.**
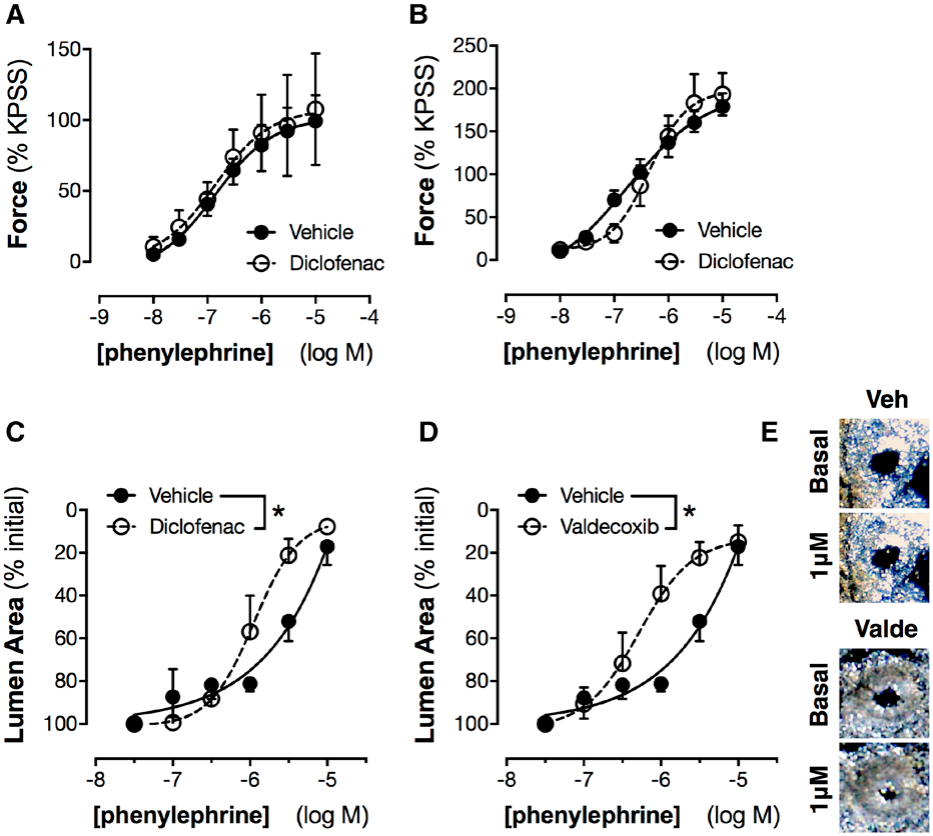
Effect of cyclooxygenase (COX) inhibition on contractile responses of aorta (**A**), renal artery (**B**), and intrarenal arcuate arteries (**C**–**E**). Contractile responses to phenylephrine were studied in aorta and renal artery by wire myography with and without nonselective COX inhibition by diclofenac (3 μmol/L; **A** and **B**) and in intrarenal arcuate arteries by imaging in precision-cut kidney slices with and without diclofenac (**C**) or the selective COX-2 inhibitor valdecoxib (**D**; 3 μmol/L). Inset panels (**E**) show example images of the effect of phenylephrine (basal vs 1 μmol/L) on arcuate arteries in the absence (veh) and presence of valdecoxib (valde). Data in **A** and **B** are expressed normalized to the response produced by a high potassium physiological salt solution (KPSS). *P<0.05 by 2-way ANOVA. n=4 to 6.

Isolated blood vessels do not spontaneously develop tone. Therefore, to determine whether COX products influence vascular tone ex vivo, vessels were contracted with phenylephrine, a selective α1-adrenoceptor agonist, in the presence and absence of diclofenac, a nonselective NSAID which blocks both COX-1 and COX-2–dependent prostanoid formation. Phenylephrine induced concentration-dependent contraction of aorta (Figure 4A), renal artery (Figure 4B), and arcuate arteries (Figure 4C). In arcuate arteries, but not aorta or renal arteries, diclofenac increased the potency of phenylephrine, suggesting that, for small arteries within the kidney but not in the main renal artery or aorta, COX products functionally antagonize contraction basally. The effect of diclofenac on phenylephrine responses in arcuate arteries was reproduced with the active metabolite of parecoxib, valdecoxib (Figure 4D and 4E), but not with the COX-1 inhibitor, SC560 (Figure S2) at concentrations which produce selective inhibition of COX-2^32^ and COX-1,^33^ respectively. These data confirm that COX-2 is the active COX isoform responsible for regulation of inhibitory vascular responses in arcuate arteries and reflect analogous observations on COX-2 control of blood flow in the kidney in vivo (Figure 1). They also fit with our previous findings that neither aorta nor the large renal artery constitutively express COX-2.^15^ Regulation of renal vascular function by COX-2 may reflect both autocrine actions of vascular prostanoids and paracrine actions of prostanoids produced by nonvascular cells in the kidney, where constitutive COX-2 expression predominates in specific tubular and interstitial cell types.^12^

**Figure 5.**
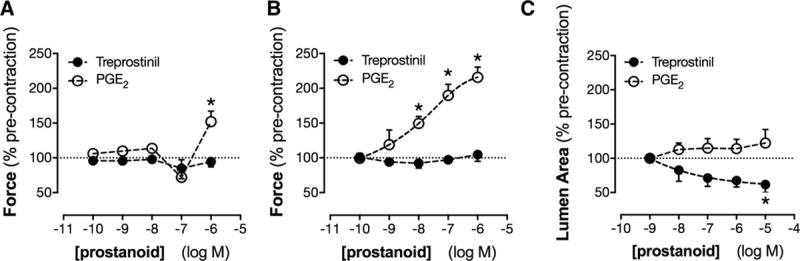
Effect of treprostinil and prostaglandin E_2_ (PGE_2_) on vascular tone in the aorta (**A**), renal artery (**B**), and intrarenal arcuate arteries (**C**). Responses to the prostacyclin analogue treprostinil and PGE_2_ were measured in precontracted vessels by wire myography (**A** and **B**) or precision-cut kidney slice imaging (**C**). *P<0.05 by 1-way ANOVA. n=4 to 6.

**Figure 6.**
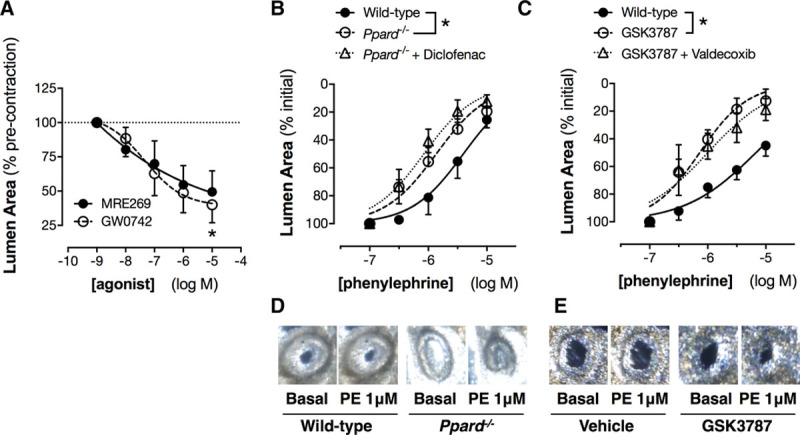
Effect of specific I-prostanoid and PPARβ/δ (peroxisome proliferator-activated receptor-β/δ) agonists (**A**) and PPARβ/δ gene deletion (**B** and **D**) or pharmacological blockade (**C** and **E**) on responses of intrarenal arcuate arteries. Responses to the I-prostanoid agonist MRE269 and to the PPARβ/δ agonist GW0742 were measured in precontracted arcuate arteries studied by precision-cut kidney slice imaging. Contractile responses to phenylephrine in intrarenal arcuate arteries were measured in precision-cut kidney slices from wild-type and PPARβ/δ knockout mice, with or without diclofenac (**B**, 3 μmol/L) and in wild-type kidney slices preincubated with the selective PPARβ/δ antagonist, GSK3787 (3 μmol/L), with or without valdecoxib (**C**, 3 μmol/L). Inset panels show example images of the effect of phenylephrine (PE; basal vs 1 μmol/L) on arcuate arteries in wild-type and PPARβ/δ knockout mice (**D**) or in wild-type vessels pretreated with GSK3787 (**E**). *P<0.05 by 1-way ANOVA (**A**) or 2-way ANOVA (**B**). n=4 to 10.

PGE_2_ and prostacyclin are the most abundant COX products produced by renal tissue.^34^ Both mediators can vasodilate some but not all vessels. Isolated renal medulla and renal cortex, as well as aorta, produced ≈4- to 5-fold more prostacyclin compared with PGE_2_ (Figure S3). However, neither PGE_2_ nor the prostacyclin analogue treprostinil relaxed the aorta (Figure 5A) or the main renal artery (Figure 5B). Indeed, PGE_2_ contracted both vessels. This is consistent with what is known about prostanoid signaling in large vessels, where the balance of vasodilator (IP, EP2, EP4) and vasoconstrictor (EP1, EP3, TP) prostanoid receptors is such that responses to prostanoid vasodilators are minimal.^35^ In contrast, in arcuate arteries, PGE_2_ had no effect while treprostinil produced clear and consistent dilator responses (Figure 5C). Treprostinil, as with authentic prostacyclin, activates 2 receptors: (1) the cell surface IP linked to adenylate cyclase and cAMP signaling and (2) the nuclear receptor PPARβ/δ linked to both genomic and nongenomic pathways.^11,36^ When measured by quantitative polymerase chain reaction, the genes encoding IP and PPARβ/δ were both expressed throughout the kidney, with PPARβ/δ being more abundant (Figure S3). To understand how activation of these receptors contributes to renal vasomotor responses, we used 2 selective agonists: MRE269 that activates IP receptors and GW0742 that activates PPARβ/δ receptors. Both drugs produced vasodilation of arcuate arteries (Figure 6A) but, as with treprostinil, neither had effects in aorta or the main renal artery (Figure S4).

### Role of Specific Prostacyclin Receptors in Renal Vascular Responses

To better understand the relative contribution of IP and PPARβ/δ pathways to endogenous COX-2–dependent renal blood flow regulation in vivo, we measured cAMP levels in the renal medulla of mice treated with parecoxib as a marker of IP receptor activation. Parecoxib treatment had no effect on renal levels of cAMP (vehicle: 12.9±5.0 pmoles/g tissue; parecoxib: 14.5±3.7 pmoles/g tissue; *P*=0.81; n=6–8) or related cGMP (vehicle: 6.3±0.8 pmoles/g tissue; parecoxib: 3.9±1.1 pmoles/g tissue; *P*=0.10; n=6–8). Consequently, we reasoned that PPARβ/δ, rather than IP signaling, is the primary driver of the renal COX-2/prostacyclin dilator pathway. This idea has not previously been proposed, but there are precedents in other systems. For example, PPARβ/δ can produce acute signaling responses through nongenomic pathways, including direct association with PKCα (protein kinase C α), and transrepression of BCL-6 (B cell-lymphoma 6).^11,36,37^ PPARβ/δ agonists can also induce vasodilation in pulmonary vessels,^38^ and PPARβ/δ, rather than IP receptors, can account for other responses caused by prostacyclin drugs in fibroblasts^39^ and platelets.^37^ Furthermore, PPARβ/δ is known to be active in the kidney, where it protects against ischemia/reperfusion injury.^40^ However, its role in renal vasomotor responses to endogenous COX-driven dilator function has not previously been investigated.

To test the idea that prostanoid-mediated activation PPARβ/δ is responsible for renal arcuate artery dilation, we compared responses of arcuate arteries in kidney slices from wild-type and PPARβ/δ-deficient mice (*Ppard*^−^^/−^; Figure 6B). Consistent with our hypothesis, phenylephrine was more potent for contraction of arcuate arteries in kidney slices from mice lacking PPARβ/δ than those from wild-type mice. This was validated pharmacologically, with a similar potentiation of the responses to phenylephrine observed when arcuate arteries in kidney slices from wild-type mice were pretreated with the highly selective PPARβ/δ antagonist, GSK3787 (Figure 6C). In addition, when PPARβ/δ function was disrupted by either genetic deletion or treatment with GSK3787, responses were not further enhanced by blocking endogenous COX-2 activity (with diclofenac or valdecoxib), suggesting that PPARβ/δ activation accounts for the majority of the vasodilator tone produced by constitutive COX-2 activity in these vessels (Figure 6B–6E). PPARβ/δ has previously been suggested to regulate activity of NFAT transcription factors^41^ which control renal COX-2 expression.^18^ To exclude the possibility that altered prostanoid production or receptor expression could complicate interpretation of the results, we studied renal prostacyclin and PGE_2_ levels and expression of COX-2, IP, and EP1-4 receptor mRNA levels and found no difference between *Ppard*^−/−^ and control animals (Table S2). Thus, the effect of PPARβ/δ disruption on arcuate artery contractility reflects its contribution to prostanoid sensing rather than a confounding dysregulation of the prostanoid pathway in these mice.

### Summary and Conclusions

COX-2 is expressed constitutively in multiple discrete regions of the body, including the brain, gut, and kidney. Other organs, such as the heart, show negligible expression. Within the sites of constitutive COX-2 expression we have identified, our data demonstrate the only place where local constitutively expressed COX-2 regulates blood flow is the kidney. As summarized in Figure S5, regulation of renal blood flow by COX-2 activity occurs through a vasodilator pathway involving prostacyclin acting on PPARβ/δ receptors, a pathway that to our knowledge has not previously been described. These data highlight the increasingly recognized role that renal COX-2 plays in systemic vascular protection and support the idea that the kidney rather than the systemic circulation is the major site of constitutive COX-2 expression where inhibition by NSAIDs could produce cardiovascular side effects. They also suggest that in the future, drugs that target inflammatory/oncogenic prostanoid signaling but spare the renal COX-2/PPARβ/δ might have improved cardiovascular and renal safety compared with NSAIDs. In a more general context, the findings of this study may have direct relevance to the mechanistic understanding of renal disease, where for example, a loss of COX-2, prostacyclin, or PPARβ/δ could contribute to reductions in medullary blood flow and renal ischemia. Conversely, if cancer risks can be overcome, targeting the PPARβ/δ pathway directly may offer a means to produce selective renal vasodilation and offer a potential treatment strategy for acute renal failure and other conditions characterized by excessive renal vascular tone.

### Perspectives

NSAIDs work by blocking COX-2 and are among the most widely used medicines worldwide. However, they produce serious cardiovascular side effects that have had far-reaching effects, including drug withdrawals and increased regulation, limiting development of new drugs in this class, and preventing use of COX-2 inhibitors in cancer prevention. Therefore, there is now an urgent unmet need to identify mechanistic pathways regulating these cardiovascular side effects. This study specifically identifies the kidney as being the sole anatomic site where local constitutive COX-2 drives vascular homeostasis and implicates a novel prostacyclin signaling pathway. Ultimately, this knowledge will foster development of new, safer drugs that spare protective COX-2–driven pathways in the vasculature.

## Acknowledgments

We thank Kelvin Jia Peng Chen, Yan Zhuang, Xuan Rui NG, Chenghao Liu, and Ming Keat SNG for providing *Ppard*^−^^/−^ mice and related expertise, Duncan Rodgers and Peter Fenwick for assistance with precision-cut tissue slice preparation and imaging, and Hime Gashaw for technical assistance.

## Sources of Funding

This work was supported by a British Heart Foundation Intermediate Basic Science Research Fellowship (to N.S. Kirkby, FS/16/1/31699), a Royal Society Research Grant (to N.S. Kirkby, RG150248), a British Heart Foundation project grant (to J.A. Mitchell and N.S. Kirkby, PG/16/83/32467), a Wellcome Trust Programme Grant (to J.A. Mitchell, 0852551Z108/Z), a grant from the Singapore Ministry of Education under its Singapore Ministry of Education Academic Research Fund Tier 2 (to X. Wang and W. Wahli, MOE2014-T2-1–036), and Start-Up grants from the Lee Kong Chian School of Medicine, Nanyang Technological University (to X. Wang and W. Wahli).

## Disclosures

None.

## Supplementary Material

## Novelty and Significance

### What Is New?

- We show that the kidney is essentially the only location where cyclooxygenase-2 has a vasomotor function, and using a novel technique of measuring vascular function in living kidney slices delineate a tonic prostacyclin/PPARβ/δ (peroxisome proliferator-activated receptor-β/δ)–driven renal vasodilator pathways through which this occurs.

### What Is Relevant?

- These data directly dispel the commonly held idea that cyclooxygenase-2 is expressed throughout the circulation to produce vasodilator prostacyclin and local vascular control and have direct relevance to our understanding of how cyclooxygenase-2 controls blood pressure and vascular health.

### Summary

In light of these findings, we must now look to the kidney rather than the systemic vasculature if we are to understand the cardiovascular side effects caused by nonsteroidal anti-inflammatory drugs.
