# Supplementary material for: Cyclooxygenase-2 Selectively Controls Renal Blood Flow Through a Novel PPARβ/δ-Dependent Vasodilator Pathway
Source: Hypertension. 2018 Feb 7;71(2):297–305. doi: 10.1161/HYPERTENSIONAHA.117.09906 (PMC5770101; doi:10.1161/HYPERTENSIONAHA.117.09906)
Supplement: Supplementary file 1 [file hyp-71-297-s001.docx]

**Cyclooxygenase-2 exerts selective control of blood flow in the kidney through a novel PPARβ/δ-dependent renal vasodilator pathway**

Nicholas S. Kirkby^1,2^, Walkyria Sampaio^3^, Gisele Etelvino^3^, Daniele Alves^3^, Katie L. Anders^1^, Rafael Temponi^3^, Fisnik Shala^1^, Anitha S. Nair^1^, Blerina Ahmetaj-Shala^1^, Jing Jiao^4^, Harvey R. Herschman^4^, Wang Xiaomeng^5,6,7,8^, Walter Wahli^9,10^, Robson A. Santos^3^ and Jane A. Mitchell^1^

**SUPPLEMENTARY MATERIAL**

*Corresponding Authors:* Dr Nicholas Kirkby (email: [n.kirkby@imperial.ac.uk](mailto:n.kirkby@imperial.ac.uk); tel: +44(0)2075947922) and Prof Jane Mitchell (email: [j.a.mitchell@ic.ac.uk](mailto:j.a.mitchell@ic.ac.uk); tel: +44(0)2075947922); Vascular Biology, National Heart & Lung Institute, Imperial College London, London SW3 6LY, UK.

**Supplementary Methods**

*Animals*

Experiments were performed on 8 week old male mice on a C57Bl/6 background. Wild-type mice were purchased from Charles River (UK) or the Biologic Science Institute (CEBIO, Federal University of Minas Gerais, Brazil) and used with pharmacological inhibitors for the majority of studies described. For other specific studies the following genetically modified strains were used: *Cox2*^fLuc/+^ mice^1^ which carry a firefly luciferase reporter gene knocked in to the COX-2 locus, *Pppard*^-/-^ mice^2^ which carry a global deletion of PPARβ/δ, and *Cox2*^-/-^ mice^3^ which carry a global deletion of COX-2. Each were generated and bred as previously described and where appropriate, compared to wild-type littermates from each line. *Cox2*^fLuc/+^ mice were used for COX-2 imaging studies and *Pppard*^-/-^ mice were used for measuring renal vascular responses. Neither strain carries a known renal or other developmental phenotype. *Cox2*^-/-^ mice were used only to as part of the *ex vivo* bioassay system for measuring COX inhibitory activity of plasma because their well-established renal developmental defects would confound interpretation of their renal vascular responses.

All animals were housed with 12 hr light/dark cycles with free access to food and water. All animal procedures were performed in line with EU directive 2010/63/EU and the NIH Guide for the Care and Use of Laboratory Animals (NIH Publication No. 85-23, revised 1996) after local ethical approval by Imperial College Animal Welfare Ethical Review Panel (license no. 70/8422), Federal University of Minas Gerais Animal Care Committee, University of California Los Angeles Animal Research Committee (protocol no. 1999-066-32) or the Nanyang Technological University and SingHealth Institutional Animal Care and Use Committees in Singapore (IACUC SHS-868).

*Microspheres*

Regional blood flow was measured in mice using a microsphere deposition technique^4, 5^ modified as we have previously described^6^ to determine the acute effects of COX-2 inhibition. Blood flow was studied before, and 20 mins are administration of the COX-2 inhibitor, parecoxib (5mg/kg i.v.; Pfizer, USA). This time point was carefully selected as, after i.v. administration, this is sufficient time to ensure the drug was distributed around the body and equilibrated with COX-2 enzymes to produce acute inhibition of COX-2-derived prostanoid production^7, 8^. More chronic dosing protocols, as well as Cox2^-/-^ mice were deliberately avoided as chronic loss of COX-2 function produces secondary effects such as changes in gene expression and vascular hormone levels and life-long loss of COX-2 in knockout mice results in profound developmental defects in the kidney. Therefore, this model of acute COX-2 inhibition was used as a way to study the effects of local, acute prostanoid production in different vascular beds, without the confounding effects of chronic blockade such as increases in circulating ADMA level and renal endothelin gene expression impacting on results.

Mice were anaesthetised with urethane (1.2g/kg i.p.; Sigma, Germany). The left femoral artery and vein were cannulated for blood withdrawal and drug administration, respectively. The right femoral artery was cannulated for measurement of blood pressure and heart rate (Biopac Systems Inc, USA), and the left cardiac ventricle cannulated via the left carotid artery for administration of microspheres. Once instrumented, mice were left to stabilise for 10 mins, before administration of yellow-green 15μm polystyrene microspheres via the left ventricle (60,000; Life Technologies, UK). Simultaneously, a reference arterial blood sample was drawn at a constant rate by syringe pump. Mice were then treated with parecoxib (5mg/kg i.v.; Pfizer, USA) and after 20 mins, crimson 15μm polystyrene microspheres (60,000; Life Technologies, UK) were administered and another reference blood sampled taken as before. Animals were next killed by cervical dislocation and tissues of interest dissected for analysis. Tissues were dissolved in ethanolic KOH (4M, 50°C; Sigma, Germany), microspheres isolated by centrifugation and the fluorescence extracted in ethyl acetate (Sigma, Germany). The fluorescence intensity of the extract was measured by fluorimetry (Cary Eclipse, Varian, Australia) and blood flow calculated by comparison to the appropriate reference blood sample according to the formula:

*Tissue blood flow (ml/min/mg) = (Fluorescence of tissue) / (Fluorescence extracted from 1ml blood withdrawn at 1ml/min) * Mass of tissue*

To confirm that repeat sampling in the same animals did not influence blood flow measurement, in a separate study, blood flow was measured before and after saline administration. Results presented in Table S1 confirm that repeat measurement had no effect of blood flow in any tissue except the stomach.

*COX-2 expression using luciferase imaging*

Bioluminescent imaging from *Cox2*^fLuc/+^ tissue was performed as previously described^9^. These animals have the coding region of the firefly luciferase gene knocked in to the *Ptgs2* locus such that luciferase is produced under the control of the native *Ptgs2* promoter. Measuring COX-2 using this reporter mouse strain is more sensitive than detection of COX-2 expression using PCR and antibody-based techniques, allows accurate quantification of relative expression without confounding effects of RNA/protein extraction and retains the spatial distribution of COX-2^1, 9^. Mice were killed by cervical dislocation, tissues of interest were dissected and bathed in D-luciferin solution (15mg/ml; PerkinElmer, USA) for 30 seconds then immediately imaged over 3 mins using a IVIS imaging system (Xenogen, USA). Where indicated, solid tissues were bisected along their midline using a scalpel blade and arranged so that the cut surface was exposed during imaging. Image data was analysed using Living Image software (Xenogen, USA) and quantified as peak photon emission per tissue – because this is independent of total tissue amount, it allows comparison between tissues of different sizes.

*Ex vivo COX activity assays*

The effectiveness and selectivity of parecoxib administered *in vivo* was assessed by *ex vivo* bioassay of plasma^10^. Because COX-2 activity in the body is scarce and so the effects of COX-2 inhibitors on prostaglandin production *in vivo* difficult to measure, we have previously established an *ex vivo* assay where the ‘COX inhibitory activity’ of plasma can be assessed^10^. Simply, this determines whether levels of a drug in the plasma after *in vivo* dosing are sufficient to produce COX-1/COX-2 inhibition when applied to test systems outside the body. Although the drug used here, parecoxib is a selective COX-2 inhibitor, we also studied effects on COX-1 because the COX-1/COX-2 selectivity of all NSAIDs is relative, and we wanted to exclude the possibility that the dose used was also producing inadvertent COX-1 inhibition which would have confounded the interpretation of the data.

To do this, plasma from mice treated with parecoxib *in vivo* as above was incubated *ex vivo* with two test systems. The first was segments (2x2x2mm) of Cox2^-/-^ mouse lung – this is a system where only COX-1 is expressed (because COX-2 has been deleted). The second was J774 murine macrophage cells (ATCC, USA) treated with LPS (24 hrs; 10µg/ml; Sigma, UK) - this is a system where only COX-2 is expressed because it is induced by the LPS. Plasma was incubated with test systems for 30 mins before stimulation with A23187 Ca^2+^ ionophore (30μM; Sigma, UK), which activates phospholipase A_2_ and ensures a replete supply of arachidonic acid is available for COX enzymes. After a further 30 mins, supernatant was removed for measurement of PGE_2_ levels by immunoassay (Cisbio, France). This assay time is sufficient to allow the activity of COX enzymes to be measured but short enough that no confounding effects on COX gene expression are likely to occur.

*Tissue PGE_2_, prostacyclin, cAMP and cGMP measurement*

Levels of PGE_2_, prostacyclin (as its spontaneous breakdown product; 6-keto-PGF_1α_) cAMP and cGMP were determined in homogenates of renal medulla and/or spleen of mice treated with parecoxib *in vivo* as above. Tissue was homogenised using a Precellys24 TissueLyser (Stretton Scientific, UK) in 20x volume of PBS containing diclofenac (100μM; Sigma, UK) to block prostaglandin formation during homogenisation, isobutylmethyxanthine (0.5mM; Sigma, UK) to prevent cAMP/cGMP degradation during homogenisation and a protease inhibitor cocktail (1X; Roche Applied Bioscience, UK) to prevent general protein breakdown. The homogenates where then separated by centrifugation and the levels of PGE_2_ (Cisbio, France), cAMP (Cisbio, France), cGMP (Cisbio, France) and 6-keto-PGF_1α_ (Enzo Lifescience, USA) were measured in the supernatant by immunoassay.

*Myography*

Thoracic aorta and renal artery were carefully dissected and cleaned of peri-adventitial material. 2mm rings of each vessel were mounted in organ baths of an isometric wire myograph (Danish Myo Technology, Demark). Vessels were bathed in Krebs buffer bubbled with 95% O_2_/5% CO_2_ and heated to 37°C and a resting tension of 6-8mN applied. To determine the contribution of endogenous COX activity to vascular responsiveness, vessels were incubated with diclofenac (3μM, 30 mins; Sigma, UK) to block all COX-1 and COX-2 activity and therefore remove all endogenous prostanoid production. To do this, it was necessary to apply phenylephrine (10nM-10μM; Sigma, UK) to the vessels to produce a contractile response as isolated vessels do not spontaneously develop tone. In the same vessels, the effect of endogenous prostaglandins was then measured. For this protocol, vessels were washed and treated with diclofenac to block endogenous prostanoids before being pre-contracted with an EC_50_ concentration of phenylephrine. Cumulative responses were then recorded to the EP receptor agonist, PGE_2_ (Sigma, UK), the mixed IP/PPARβ/δ agonist prostacyclin-mimetic, treprostinil (Cayman Chemical, USA), the selective IP agonist, MRE269 (Cayman Chemical, USA) or the selective PPARβ/δ agonist, GW0742 (R&D Systems, UK). Responses were measured using LabChart 4 software (AD Instruments, UK). Responses to exogenous prostanoid drugs were normalised as a percentage of the pre-existing contraction as is convention for dilator responses.

*Tissue slice preparation and imaging of vascular responses*

Vascular responses in precision cut tissue slices were measured essentially as we have previously performed in lung^11, 12^. Precision-cut slices of intact mouse kidney (~150μm thick) were prepared using a Krumdieck Tissue Slicer (Alabama Research, USA). Kidney slices were left to equilibrate at 37°C for 1 hour during which arcuate arteries were identified by microscopy based on their location at the boundary of cortex and medulla and relationship with attached veins. To measure vascular responses, in slices bathed in Krebs buffer at 37°C, individual arteries were focused at 5X objective magnification under a video microscope (Zeiss, Germany). To determine the contribution of endogenous COX-1- and COX-2-derived prostanoids and the PPARβ/δ receptor in vascular responses, slices were pre-treated with non-selective COX-1/COX-2 inhibitor, diclofenac (3μM), the selective COX-2 inhibitor, valdecoxib (3μM; Sigma, UK) the selective COX-1 inhibitor, SC560 (100nM; Sigma, UK) and/or the selective PPARβ/δ antagonist GSK3787 (3μM; Sigma, UK) for 30 mins and then cumulative response curves to phenylephrine measured (30nM-10μM). To determine the sensitivity of these vessels of exogenous prostanoids, in separate slices, after treatment with diclofenac to block all endogenous COX activity, arteries were pre-contracted with an EC_50_ concentration of phenylephrine, and cumulative responses to PGE_2_ (EP receptor agonist) treprostinil (mixed IP/PPARβ/δ agonist prostacyclin-mimetic), MRE269 (selective IP agonist) or GW0742 (selective PPARβ/δ agonist) measured. Vessel diameter was quantified by tracing their outline in single video frames using ImageJ software (NIH, USA). Constrictor responses were normalised as a percentage of the initial vessel diameter and dilator responses as a percentage of the pre-contraction as is convention of vascular response data.

*Prostaglandin release*

To measure prostaglandin levels from renal and aortic tissue where it is not possible to obtain comparable homogenates due to the differences in tissue volumes, intact segments of tissues segments (2x2x2mm) were used. Tissue pieces were placed DMEM media (Sigma, UK) containing A23187 Ca^2+^ ionophore (30μM) for 30 mins at 37°C. A23187 activates phospholipase A_2_ and in so doing ensures that there is replete arachidonic acid available and removes this as a limiting factor for observing the relative COX activity and prostaglandin production. PGE_2_ and prostacyclin (measured as 6-keto-PGF_1α_) were measured in the conditioned media by immunoassay (Cayman Chemical, USA). This method only applies to the data in Figure S3 as in all other figures, prostanoids were measured in tissue homogenates without stimulation.

*qPCR*

Gene expression in renal and aortic tissue was measured by quantitative reverse-transcriptase PCR. RNA was extracted from tissue homogenates using an RNeasy Mini kit (Qiagen, UK) and converted to cDNA using reverse transcriptase (Fermantas, UK) and oligo(dT) primers (Life Technologies, UK). Expression of *Ptgir* (IP receptor; probe ID: Mm00801939_m1), *Ptger1* (EP1 receptor; probe ID: Mm00443098_g1), *Ptger2* (EP2 receptor; probe ID: Mm00436051_m1), *Ptger3* (EP3 receptor; probe ID: Mm01316856_m1) *Ptger4* (EP4 receptor; probe ID: Mm00436053_m1) and *Ppard* (PPARβ/δ; probe ID: Mm00803184_m1) were then measured using TaqMan gene expression assays (Life Technologies, UK), qPCR master mix (Fermenstas, UK) and an Applied Biosystems (UK) 7500 Fast instrument. Data were normalised to expression of housekeeping genes *18s* (probe ID: Mm0392899_g1) and *Gapdh* (probe ID: Mm99999915_g1) using the comparative C_T_ method.

*Statistics and data analysis*

Data are presented as means ± standard errors for n experiments. Myography and vessel imaging data sets were normalised as described above. All data were statistically analysed using Prism 7 software (Graphpad Software, USA) as defined in individual figure legends – typically either Student’s unpaired t-test for *in vivo* data or two-way ANOVA for vascular response data. Differences were considered statistically significant where p<0.05.

*References for supplementary methods*

1. Ishikawa TO, Jain NK, Taketo MM, Herschman HR. Imaging cyclooxygenase-2 (cox-2) gene expression in living animals with a luciferase knock-in reporter gene. *Mol Imaging Biol*. 2006;8:171-187

2. Nadra K, Anghel SI, Joye E, Tan NS, Basu-Modak S, Trono D, Wahli W, Desvergne B. Differentiation of trophoblast giant cells and their metabolic functions are dependent on peroxisome proliferator-activated receptor beta/delta. *Mol Cell Biol*. 2006;26:3266-3281

3. Morham SG, Langenbach R, Loftin CD, Tiano HF, Vouloumanos N, Jennette JC, Mahler JF, Kluckman KD, Ledford A, Lee CA, Smithies O. Prostaglandin synthase 2 gene disruption causes severe renal pathology in the mouse. *Cell*. 1995;83:473-482

4. Prinzen FW, Bassingthwaighte JB. Blood flow distributions by microsphere deposition methods. *Cardiovasc Res*. 2000;45:13-21

5. Rudolph AM, Heymann MA. The circulation of the fetus in utero. Methods for studying distribution of blood flow, cardiac output and organ blood flow. *Circ Res*. 1967;21:163-184

6. Botelho-Santos GA, Bader M, Alenina N, Santos RA. Altered regional blood flow distribution in mas-deficient mice. *Ther Adv Cardiovasc Dis*. 2012;6:201-211

7. Ahmetaj-Shala B, Kirkby NS, Knowles R, Al'Yamani M, Mazi S, Wang Z, Tucker AT, Mackenzie L, Armstrong PC, Nusing RM, Tomlinson JA, Warner TD, Leiper J, Mitchell JA. Evidence that links loss of cyclooxygenase-2 with increased asymmetric dimethylarginine: Novel explanation of cardiovascular side effects associated with anti-inflammatory drugs. *Circulation*. 2015;131:633-642

8. Kirkby NS, Lundberg MH, Harrington LS, Leadbeater PD, Milne GL, Potter CM, Al-Yamani M, Adeyemi O, Warner TD, Mitchell JA. Cyclooxygenase-1, not cyclooxygenase-2, is responsible for physiological production of prostacyclin in the cardiovascular system. *Proc Natl Acad Sci U S A*. 2012;109:17597-17602

9. Kirkby NS, Zaiss AK, Urquhart P, Jiao J, Austin PJ, Al-Yamani M, Lundberg MH, MacKenzie LS, Warner TD, Nicolaou A, Herschman HR, Mitchell JA. Lc-ms/ms confirms that cox-1 drives vascular prostacyclin whilst gene expression pattern reveals non-vascular sites of cox-2 expression. *PLoS One*. 2013;8:e69524

10. Armstrong PC, Kirkby NS, Zain ZN, Emerson M, Mitchell JA, Warner TD. Thrombosis is reduced by inhibition of cox-1, but unaffected by inhibition of cox-2, in an acute model of platelet activation in the mouse. *PLoS One*. 2011;6:e20062

11. Moreno L, Perez-Vizcaino F, Harrington L, Faro R, Sturton G, Barnes PJ, Mitchell JA. Pharmacology of airways and vessels in lung slices in situ: Role of endogenous dilator hormones. *Respir Res*. 2006;7:111

12. Faro R, Moreno L, Hislop AA, Sturton G, Mitchell JA. Pulmonary endothelium dependent vasodilation emerges after birth in mice. *Eur J Pharmacol*. 2007;567:240-244

| **Tissue/Region** | **Blood flow (ml/min/g)** | | **p** |
| --- | --- | --- | --- |
|  | **Basal** | **Saline** |  |
| Skeletal Muscle | 0.3 ± 0.0 | 0.3 ± 0.2 | 0.81 |
| Bone | 0.4 ± 0.1 | 0.7 ± 0.4 | 0.28 |
| Hippocampus | 0.5 ± 0.1 | 0.6 ± 0.1 | 0.61 |
| Thymus | 0.5 ± 0.1 | 0.8 ± 0.3 | 0.27 |
| Trachea | 0.5 ± 0.1 | 0.4 ± 0.1 | 0.47 |
| Skin | 0.5 ± 0.1 | 0.7 ± 0.2 | 0.43 |
| Stomach | 0.6 ± 0.1 | 0.1 ± 0.0 | 0.02 * |
| Testes | 1.3 ± 0.2 | 1.3 ± 0.1 | 1.00 |
| Bladder | 1.3 ± 0.2 | 0.9 ± 0.3 | 0.32 |
| Adipose Tissue | 1.5 ± 0.4 | 2.0 ± 0.2 | 0.45 |
| Mesentery | 1.9 ± 0.3 | 2.0 ± 0.3 | 0.86 |
| Ileum | 2.0 ± 0.3 | 2.6 ± 0.7 | 0.40 |
| Brain Stem | 2.3 ± 0.5 | 0.7 ± 0.1 | 0.06 |
| Colon | 2.4 ± 0.5 | 1.6 ± 0.4 | 0.34 |
| Renal Medulla | 2.6 ± 0.5 | 2.2 ± 0.8 | 0.63 |
| Spleen | 3.6 ± 0.8 | 2.3 ± 0.4 | 0.30 |
| Cerebellum | 4.9 ± 0.8 | 2.9 ± 0.1 | 0.21 |
| Cerebral Cortex | 5.4 ± 1.1 | 3.0 ± 0.5 | 0.21 |
| Heart | 10.6 ± 1.8 | 7.4 ± 1.0 | 0.30 |
| Renal Cortex | 10.9 ± 1.3 | 9.2 ± 1.0 | 0.45 |
| Duodenum | 13.3 ± 1.6 | 9.0 ± 1.5 | 0.13 |

**Table S1: Effect of saline bolus on regional blood flow.** Data are presented as mean ± standard error. *, p<0.05 by Student’s unpaired t-test. n=4-10.

| **Measurement** (units) | **PPARβ/δ^+/+^** | **PPARβ/δ^-/-^** | **p** |
| --- | --- | --- | --- |
| **PGE_2_** **levels** (ng/g tissue) | 3.4 ± 0.7 | 2.8 ± 0.7 | 0.53 |
| **6ketoPGF_1ɑ_ levels** (ng/g tissue) | 5.9 ± 1.2 | 4.8 ± 1.1 | 0.52 |
| ***Ptgs2* expression** (fold-difference) | 1.0 ± 0.8 | 1.1 ± 0.3 | 0.93 |
| ***Ptger1* expression** (fold-difference) | 1.0 ± 0.5 | 1.1 ± 0.2 | 0.83 |
| ***Ptger2* expression** (fold-difference) | 1.0 ± 0.6 | 0.8 ± 0.2 | 0.75 |
| ***Ptger3* expression** (fold-difference) | 1.0 ± 0.6 | 1.6 ± 0.2 | 0.31 |
| ***Ptger4* expression** (fold-difference) | 1.0 ± 0.7 | 2.0 ± 0.3 | 0.21 |
| ***Ptgir* expression** (fold-difference) | 1.0 ± 0.5 | 0.7 ± 0.1 | 0.46 |

**Table S2: Effect of PPARβ/δ deletion on prostanoid production and expression of COX-2 and prostanoid receptors in the renal medulla.** Data are presented as mean ± standard error. p values by Student’s unpaired t-test. n=3-7.


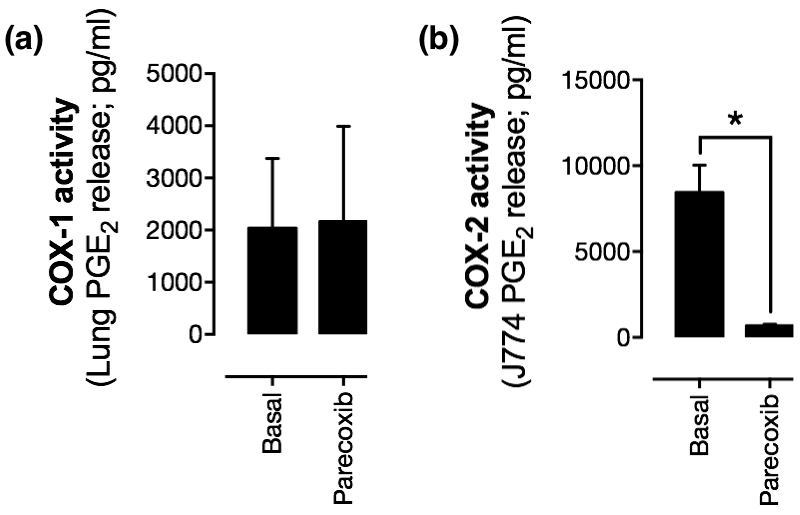


**Figure S1: Inhibitory activity of plasma from mice treated with parecoxib in vivo on COX-1 (a) and COX-2 (b) activity ex vivo.** COX-1 inhibitory activity bioassayed as PGE_2_ production by segments of lung from *Cox2*^-/-^ mice. COX-2 inhibitory activity bioassayed as PGE_2_ production from J774 murine macrophages treated with LPS (10μg/ml) to induce COX-2. Data are presented as mean ± standard error. *, p<0.05 by Student’s unpaired t-test. n=5-6.


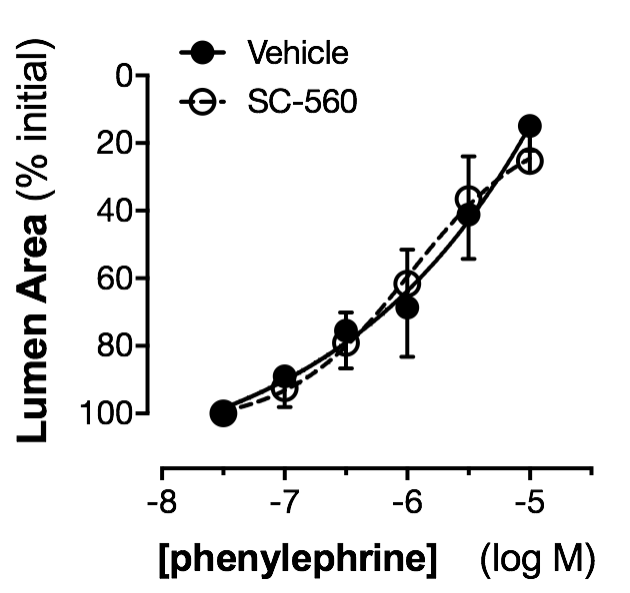


**Figure S2: Effect of selective COX-1 inhibition on contractile responses of intra-renal arcuate arteries.** Contractile responses to phenylephrine were studied in intra-renal arcuate arteries by imaging in precision-cut kidney slices after incubation with the selective COX-1 inhibitor SC560 (100nM). *, p<0.05 by two-way ANOVA. n=4-6.


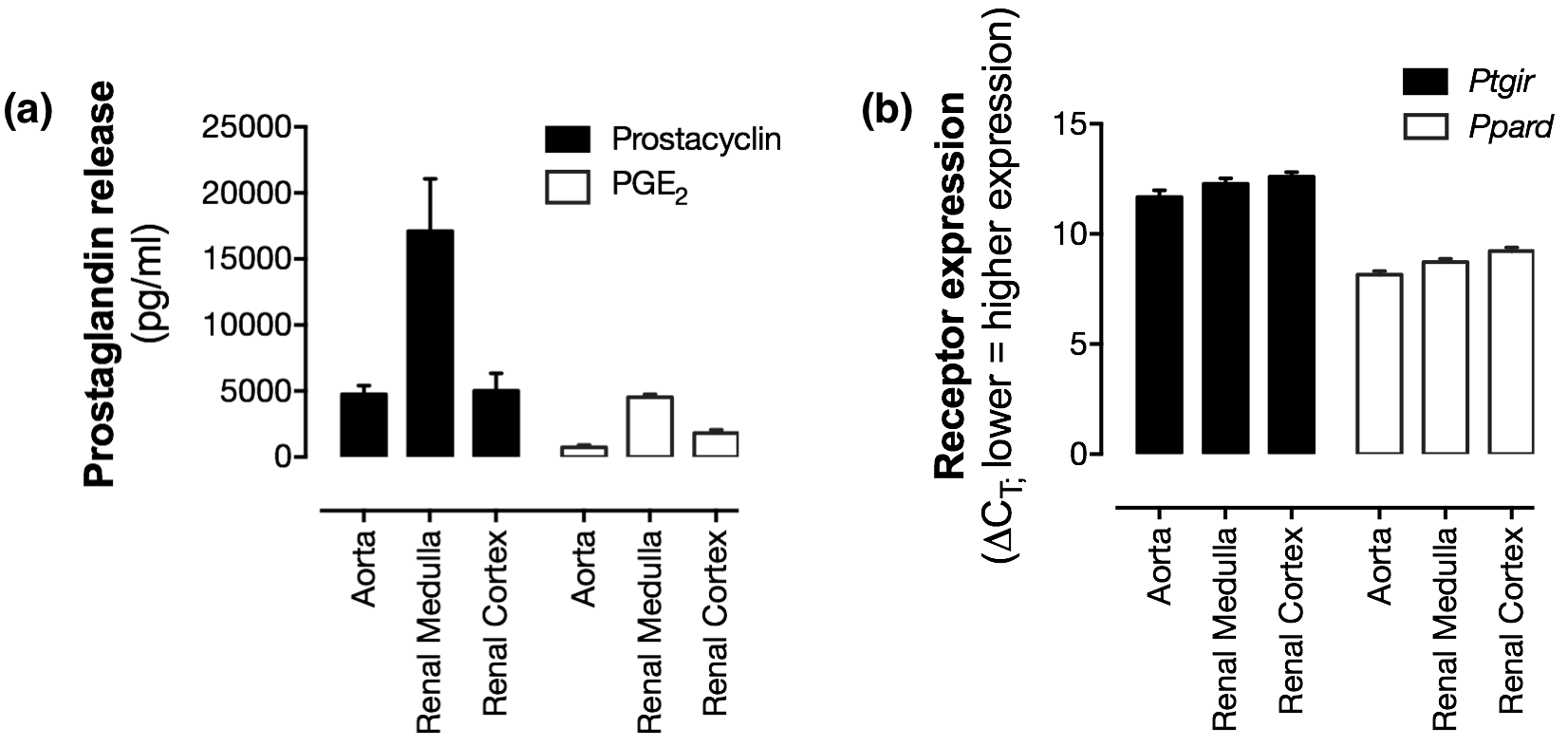


**Figure S3: Prostanoid production (a) and receptor expression profile (b) in renal and vascular tissue.** Prostacyclin and PGE_2_ production were measured by immunoassay in supernatants after incubation with intact sections of renal medulla, renal cortex or aorta. Expression of the genes encoding the IP receptor (*Ptgir*) or PPARβ/δ (*Ppard*) were measured by qPCR and expressed at ΔC_T_ after correction for 18s and Gapdh expression (lower value = higher expression). n=3-4.

**
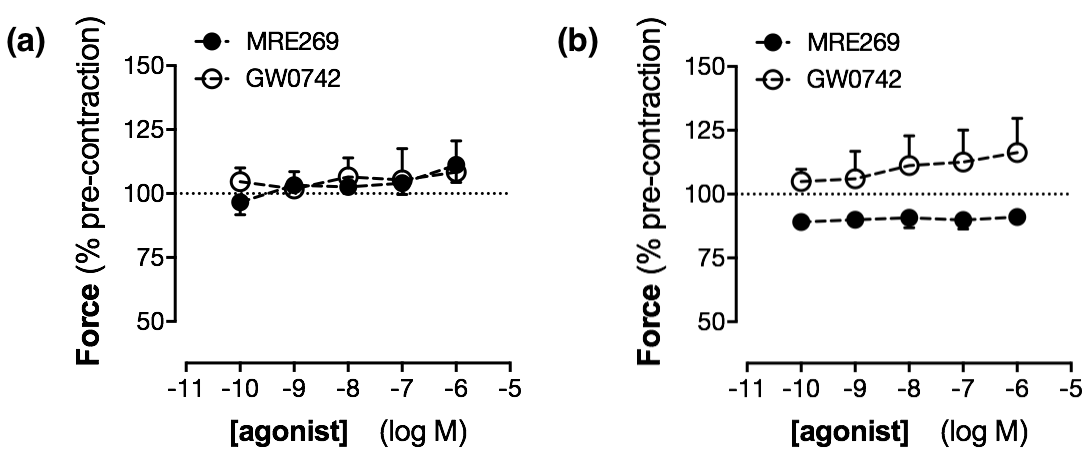
**

**Figure S4: Effect of specific IP and PPARβ agonists on vascular tone in aorta (a) and renal arteries (b).** Responses to the IP agonist MRE269 and PPARβ agonist GW0742 in phenylephrine pre-contracted aorta and renal arteries studied by wire myography. *, p<0.05 by one-way ANOVA. n=4-5.


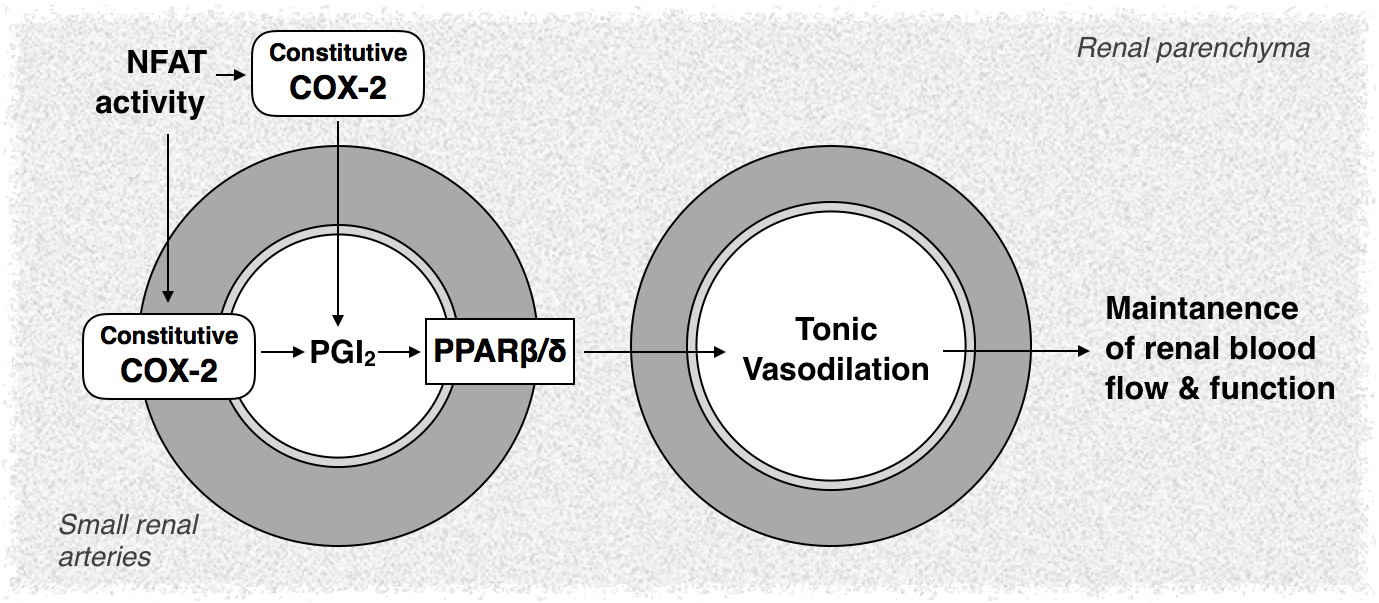


**Figure S5: Schematic for regulation of renal blood flow by NFAT-mediated constitutive COX-2 expression, prostacyclin (PGI_2_) and PPARβ/δ.**
